# Automated Prototype for *Bombyx mori* Cocoon Sorting Attempts to Improve Silk Quality and Production Efficiency through Multi-Step Approach and Machine Learning Algorithms

**DOI:** 10.3390/s23020868

**Published:** 2023-01-12

**Authors:** Simone Vasta, Simone Figorilli, Luciano Ortenzi, Simona Violino, Corrado Costa, Lavinia Moscovini, Francesco Tocci, Federico Pallottino, Alberto Assirelli, Alessio Saviane, Silvia Cappellozza

**Affiliations:** 1 Consiglio per la Ricerca in Agricoltura e l’Analisi dell’Economia Agraria (CREA), Centro di Ricerca Ingegneria e Trasformazioni Agroalimentari, Monterotondo, 00015 Rome, Italy; 2 Department of Agricultural and Forestry Sciences (DAFNE), Tuscia University of Viterbo, 01100 Viterbo, Italy; 3 Consiglio per la Ricerca in Agricoltura e l’Analisi dell’Economia Agraria (CREA), Centro di Ricerca Agricoltura e Ambiente, Laboratorio di Gelsibachicoltura, 35143 Padua, Italy

**Keywords:** reelability, defective cocoons, stained cocoons, dead/alive pupae, undersize/oversize cocoons, machine learning

## Abstract

Cocoon sorting is one of the most labor-demanding activities required both at the end of the agricultural production and before the industrial reeling process to obtain an excellent silk quality. In view of the possible relaunch of European sericulture, the automatization of this production step is mandatory both to reduce silk costs and to standardize fiber quality. The described research starts from this criticality in silk production (the manual labor required to divide cocoons into different quality classes) to identify amelioration solutions. To this aim, the automation of this activity was proposed, and a first prototype was designed and built. This machinery is based on the use of three cameras and imaging algorithms identifying the shape and size of the cocoons and outside stains, a custom-made light sensor and an AI model to discard dead cocoons. The current efficiency of the machine is about 80 cocoons per minute. In general, the amelioration obtained through this research involves both the application of traditional sensors/techniques to an unusual product and the design of a dedicated sensor for the identification of dead/alive pupae inside the silk cocoons. A general picture of the overall efficiency of the new cocoon-sorting prototype is also outlined.

## 1. Introduction

Currently, silk is an agricultural commodity, which constitutes less than 0.2% of the present market for natural textile fibers [1]. China is the silk world monopolist because, in the second part of the last century, agricultural cocoon and raw silk production were delocalized mainly to this country, permitting the cheap sourcing of raw material for the European and Japanese textile industries [2]. Nowadays, silk production in China is progressively diminishing because of the country’s industrialization and the urbanization of people, especially the youngest ones. Other problems impacting silk production are climatic changes, pollution, soil consumption, competition in the use of soil for food crops for the Chinese middle class and self-consumption of silk for the local industry; all these events affect the quantity and especially the quality of Chinese silk exports to EU [3]. The European silk industry, flourishing and very important in terms of the export of finished products, is thus facing a sourcing problem and a rise in the international silk price. This is also because the international demand for silk has been multiplying due to new applications for silk proteins (cosmetics, biomedicals, new materials) [4]. With a view to re-establishing at least a share of raw silk production in Europe, to cover a part of the silk industry demand, setting up a modern and convenient agroindustry chain is of basic importance [5]. Silk is the natural fiber produced by the silkworm *Bombyx mori* L. The insect secretes the silk filament to build a cocoon, in which the larva completes its metamorphosis. Silk thread obtainment is the result of a series of agricultural and industrial processes, among which mulberry cultivation for silkworm feeding, larval rearing from the eggs to the spinning stage, cocoon processing and reeling are the most prominent. Without any human intervention, the moth would develop inside its self-assembled silk case and eventually emerge from it by softening the protein glue (sericin), which makes the cocoon close-knit, through an enzymatic process. The silk industrial chain usually devitalizes the insect by hot hair at the pupal stage, while it is still in the cocoon, to avoid damages to the silk shell that can be, therefore, preserved, for a long time. Afterwards, the reeling process takes place, and the commercial silk skein is produced by unwinding the cocoon thread. To produce superior raw silk, cocoons should be assessed for their silk shell rate out of the whole cocoon, the length of the silk filament and the percentage of defective cocoons. Defects of cocoons are determined by many external factors and can be grouped into defects of cleanliness, which are the most important (inside stained cocoons/dead cocoons and outside stained cocoons) and defects of shape and texture (double cocoons, printed cocoons, malformed cocoons, flimsy cocoons, thin-end cocoons) [6]. Usually, farmers manually set up cocoon sorting at their facilities before cocoon delivery for sale and drying. In countries with advanced sericulture, cocoon lots are subjected to systematic testing and grading before sale, and prices are based on the cocoon quality [6]. The process is very long and tedious for farmers, therefore affecting production costs, and is mostly based on visual inspection and personal experience. In order to organize a modern production chain, an automated and non-subjective sorting and classification machine is required, with the twofold aim of establishing an equal price for farmers, related to the quality of their cocoon harvest, and classifying and organizing cocoons for the different branches of the processing industries mentioned above: textiles, cosmetics, biomedical, and new materials [7]. Appropriate cocoon-drying techniques, cocoon sorting to address the best quality products to reeling operations and an efficient reeling process are vital to supply good-quality silk [6]. Although new technologies are available from other agricultural fields, the silk industry has not transitioned to them because it has always relied on cheap labor costs more than on advanced methodologies of processing [8]. In recent years, a series of attempts have been recorded to apply artificial intelligence (AI) [9] to the sericultural chain from eggs to raw silk production [10]. In particular, some technologies, such as machine learning’s generative adversarial networks (GANs), could allow the planning of silkworm rearing, describing certain parameters, such as the color and shape of mulberry leaves or the classification of silkworm cocoons, from input images [9]. An important application of AI is the prediction of silk supply and demand in the long term based on export and import reports recorded over the years [9]. A further work proposed the use of image analysis techniques for the morphological examination of silkworm eggs [10]. In detail, 60 sample images of seven shades of color groups of silkworm eggs were considered. The adopted software showed 90% classification accuracy and was used for inspection purposes.

Through the combination of terahertz (THz) technology and a learning algorithm, it was possible to obtain information about the internal morphology of silkworm eggs. In fact, this algorithm showed the fast recognition of the growth stages of the silkworm embryos, presenting a recognition accuracy of 98.5% [11].

In the work by Kanjanawanishkul et al. [12], Eri silkworm pupae were examined by image analysis for their quality and size, as they are often used as protein food in Asian countries. Pupal shape and color were tested by developing two separate neural networks. After identifying deformed and discolored pupae, the remaining pupae were classified according to their size. The correct classification rates for shape and color resulted in 57% and 60% out of the total analyzed individuals. For the size, the percentage of correct classification was 94%.

A further study identified the sex of silkworm pupae using radial-based neural networks (RBF-NNs), through which an accuracy rate of more than 98% was achieved [13].

In addition to using artificial intelligence models to discriminate live cocoons from dead cocoons, there are also some examples in the literature of work using near-infrared spectroscopy for such discrimination. In fact, in the study by Lee et al. [14], live cocoons were discriminated from dead cocoons by near-infrared spectroscopy. A total of 367 normal cocoons and 152 dead cocoons were considered in order to determine whether they were alive or dead. Near-infrared transmission spectra were obtained for each cocoon in the 900–1700 nm wavelength band. Therefore, the discrimination showed a discriminative accuracy of 94.80%. Another work on discrimination between live and dead worms on the basis of sex using near-infrared spectroscopy was carried out by Zhu et al. [15]. The high-speed sex discrimination of live silkworm pupae was successfully achieved by combining the data with SIMCA analysis. The results indicated that this method achieved a 98.0% correct identification rate. 

The technological application described in the present study and based on the patent by Assirelli et al. [16] attempts to solve this criticality of the sericultural process and to establish a step forward in the mechanization of silk production by setting up an automated prototype of a sorting machine.

## 2. Materials and Methods

### 2.1. Silkworm Samples

Cocoon samples were produced by different farmers collaborating on a demonstration activity carried out in the framework of the Serinnovation project (Rural Development Program 2014–2020 of the Veneto Region). Briefly, silkworm eggs of the polyhybrid strain [(129 × 127) × (125 × 126)] were produced at the CREA–AA, Sericulture Laboratory of Padua, where they hatched. Then, the larvae were reared until the third instar at a collective nursery farm and distributed to different producers for the rearing of the fourth and fifth instars and commercial cocoon spinning. After harvesting, cocoons were manually sorted into different categories (from the first to the third grade), according to their shape, dimensions, and color, by the same farmers and, thereafter, brought to CREA for drying. After the drying process, different cocoon samples were prepared by randomly picking cocoons from the different producers and dividing them into the following categories:

- 100 cocoons with a shape fit for reeling: mixed stained and white, and mixed alive or dead pupae inside;
- 100 cocoons with a shape not fit for reeling: mixed stained and white, and mixed alive or dead pupae inside;
- 100 cocoons with a shape fit for reeling: stained, mixed alive or dead pupae inside;
- 100 cocoons with a shape fit for reeling: white, mixed alive or dead pupae inside;
- 100 cocoons with a shape fit for reeling: mixed stained and white, with alive pupae inside;
- 100 cocoons with a shape fit for reeling: mixed stained and white, with dead pupae inside.

### 2.2. The Sorting Machine Prototype

The prototype was based on an opto-electronic machine and was conceived to operate through three main sections using three cameras and one light sensor. The selection parameters were set as follows:

- Shape and size, using a camera and imaging algorithms;
- Outside stains, using two opposite-direction-mounted cameras and imaging algorithms;
- Amount of light passing through, using a custom-made light sensor and AI model.

**Figure 1:**
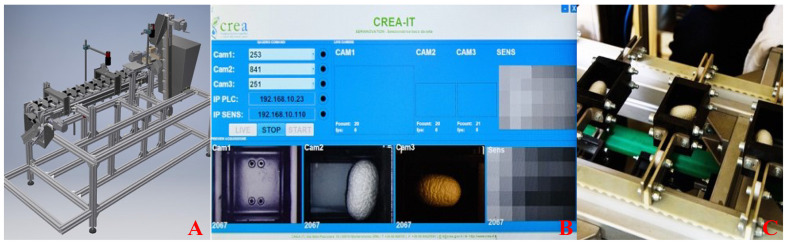
(**A**) 3D CAD render of the prototype, (**B**) screenshot of GUI written in Java, (**C**) custom-made conveyor belt.

This sorting machine (Figure 1A) was built around a conveyor belt moved by one stepper motor, and all the mechanical movement systems were controlled by a programmable logic controller (PLC). 

**Figure 2:**
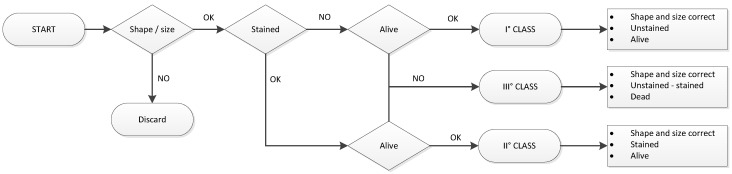
Flow chart of the sorting and selection steps of the sorting machine.

Furthermore, pneumatic actuators for the final and initial selections were activated by solenoid valves also controlled by the same PLC as above (Figure 2).

The three cameras installed on the prototype were connected to a main personal computer (PC) via one ethernet cable each, passing through a power over ethernet (PoE) switch, and they received trigger signals to take pictures at a certain time. Preliminary operations done prior to the acquisition process ere made with the graphical user interface (GUI) designed and managed in the Java programming language; furthermore, using the same GUI, it was possible to monitor various parameters, such as the number of photos taken, status of the cameras, status of the analog to digital converter (ADC), and so on, or doing other operations like starting or stopping the acquisition (Figure 1B).

All these tasks on the main PC were achieved using multithreading programming techniques, and they were performed while storing at the same time all the synced images from the cameras in addition to the raw data from the matrix sensor. Syncing between PC, custom light sensor and PLC was carried out with the Modbus ethernet communication protocol, which was implemented on the Arduino Portenta H7 using an appropriate software library.

The hierarchy we chose, based on the Modbus communication protocol, can be summarized by establishing one client represented by the PC and two servers, represented by the Arduino and the PLC. Between the PC and the PLC, there was a Boolean type of data exchange, which was conceived to coordinate the movement of all the mechanical parts. On the other hand, a correlation between the light sensor and the PC was built to allow Boolean data to handle the timing of acquisition, thus later using the same bus to pass raw data to the PC’s ModBus registers.

The whole mechanical construction was made possible by the extensive use of aluminum profiles, enabling a high level of modularity without compromising the structural strength. The conveyor belt was based upon an unusual design, and for this reason, a custom-made model manufactured in Italy was used in this project (Figure 1C). 

The prototype was built to operate on a horizontal type of motion, starting from a vertical feeder that catches and transports silkworm cocoons towards a 45 degree slide. The first camera, placed at the end of the slide, was used to discard cocoons whose shape was not adequate by the action of two pneumatic actuators mounted perpendicularly to the camera. Cocoons that were considered shape-approved fall by gravity into specifically designed small capsules fixed to a horizontal conveyor belt. Using this conveyor belt, the small capsule with one cocoon inside advanced to the next two cameras, which were specialized in stain selection. 

These cameras were the same model as the shape-selection one, while the difference was in the software used to determine this parameter and in the placement. In fact, one camera faced the cocoon from below and the other from above; this arrangement was used to frame two different faces of the same sample, thus reducing any possible reading error, such as mistaking the state of the cocoon. The last selection station was dedicated to the discrimination of alive vs. dead pupae inside the cocoons and was made by a complex sensor based on photodiodes for which a custom PCB and software were designed. After all the processing was done, the machine could send individual samples to different containers according to the different quality grades through a pneumatic-oriented slide placed at the end of the conveyor belt.

The prototype applied different approaches for the detection of the selection traits.

### 2.3. Sorting for Defective Cocoon Shape and Size

**Figure 3:**
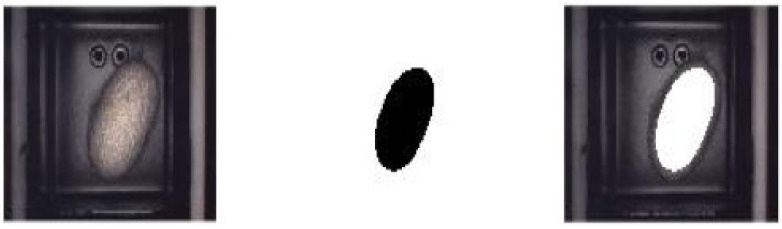
Result of the cocoon segmentation procedure. From left to right: image of cocoons, segmented image and superimposition of the segmented cocoon outline with the original image.

This step consisted of a station where cocoons were selected according to their shape. A digital camera was placed about twenty centimeters above the cocoon resting space, and in between, there was a light-emitting diode (LED) light made by Banner, model LEDWO50M, which could project white light on-axis with the camera lens to produce even lighting on the surface of the object. Images were acquired by an optoelectronic system based on a GigE Vision Camera Manta G-504B/C with a 5 megapixel (MP) Sony ICX655 RGB sensor operating at a resolution of 1292 × 964 pixels. Cocoon shape and size were extracted through an image analysis protocol (Figure 3).

Cocoon outlines were extracted by segmenting the original images in the following way. The first image’s HSB coordinates were extracted, and the matrix M was obtained as:

M = log(|rgb2gray(H,S,B)|)

With rgb2gray(H,S,B) = 0.2989 × H + 0.5870 × S + 0.1140 × B. Note that this is the same linear combination used to obtain the luminance from the RGB colorimetric space along with the ITU-R Recommendation BT.601.

The HSB color space was chosen because it is very efficient in emphasizing the edges in pictures. In particular, the B component simulated the change in illumination from a white source, which was, of course, enhanced across the edges of an object. The H and S components were used to distinguish the inner side of the border from the other one. They indeed changed significantly going from the cocoon to the background.

The obtained matrix M was finally normalized to the maximum, and the result transformed to uint8 format and binarized using the Otsu threshold [17]. 

After binarization, a total of 180 points (x, y) equally angularly spaced (one point every π/90 rad) from the centroid were digitized along the outline of 1006 cocoons. Coordinates were aligned by generalized Procrustes analysis [18]. In order to extract synthetic features for feeding a binary classifier (properly-shaped cocoons), the 180 aligned coordinates (i.e., the overall shape) of each cocoon were analyzed by elliptic Fourier analysis (EFA) [19]. Cocoon-shape outline could be approximated by a polygon of x–y coordinates. EFA is based on the separate Fourier decompositions of the incremental changes of the x and y coordinates as functions of the cumulative chordal length of the outline polygon. It yields to the spectrum of the cocoon-shape closed contour in terms of harmonically related trigonometric curves. For each harmonic equation, two Fourier coefficients were computed for both the x and y projections, thus the total number of coefficients was 4n, where n was the number of harmonics fitted to the outline [20]. The total number of harmonics that can be computed for any outline is equal to half of the total number of outline coordinates (the “Nyquist frequency”). The Fourier series was truncated at the value of k, at which the average cumulative power is 99.999% of the average total power [19]. For any outline, the total power was calculated as the sum, from 1 to k, of individual harmonic powers where k is equal to the Nyquist frequency [21]. The harmonic coefficients describe the size, shape, and orientation of each harmonic ellipse and form the input to multivariate statistics. The outline extraction and EFA procedure was conducted using Matlab (rel 7.1; Mathworks, Natick, MA, USA). The shape selection of cocoons was done using logistic regression with the machine learning algorithm taking the Z-score of the EFA coefficients as input variables.

The Z-score of a variable x is defined as (X-m)/s where m is the mean value of x and s is its standard deviation. The Z-score is needed to compare variables having very different support.

Given the extreme sample imbalance (81% well-shaped cocoons and 19% bad-shaped cocoons) of the original data, a new dataset of 356 samples was built, joining together the 178 bad-shaped cocoons and 178 well-shaped cocoons randomly selected among the 773 well-shaped cocoons belonging to the original dataset.

The obtained sample dataset was divided into two subsets: the training (70%) and the test (30%) set. This ratio results indeed in the best training-to-testing trade off with respect to overfitting and underfitting. The results were validated through cross validation, averaging over 100 iterations on the whole original dataset of 951 cocoons.

Size was measured by counting the pixels belonging to the cocoon outline and the whole cocoon selected according to a threshold criterion. Cocoons with areas smaller and larger than a fixed value, therefore, were discarded.

### 2.4. Sorting for Outside Stained Cocoons

For the stain-selection phase, two cameras were placed about twenty centimeters apart from the moving slot. The first camera framed the cocoon from above, giving an image of the top part.

**Figure 4:**
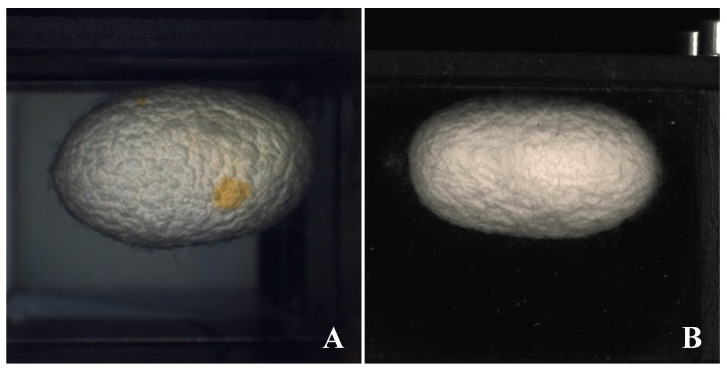
Images taken from the top camera (**A**) and bottom camera (**B**). The first picture shows evident staining (orange spot) that is not observable in the second camera acquisition.

Subsequently, the second camera instead framed the same cocoon from below, therefore giving an image that represented the bottom part. Considering that spots can occur on the opposite side with respect to that examined by the camera, acquiring and labelling two images from two different viewpoints led to a better representation of the cocoon surface. Images were acquired by two optoelectronic systems, both based on a GigE Vision Camera Manta G-504B/C with a 5 megapixel (MP) Sony ICX655 RGB sensor operating at a resolution of 1292 × 964 pixels. The first stain-selection camera used a LED light made by Banner, model LEDWO50M, while the second camera used two warm white LED strips powered at 24 V and mounted in parallel to the conveyor-belt direction of motion (Figure 4).

Given the emergent difference in the color of stains with respect to the cocoon surface, to observe the presence of stains outside the cocoons, an empirical imaging procedure was applied. A fixed threshold approach was implemented on the HSB colorimetric space.

In order to sort the stain-free cocoons, images were filtered with two sets of threshold values on the HSB colorimetric space; these values were used according to the different illumination conditions and are reported in Table 1.

If the filtered image contains objects with an area larger than 144 pixels^2^, it means that the cocoon presents stains. The percentages of correct classification for both top- and bottom-camera positions were then extracted.

### 2.5. Sorting for Dead Cocoons

**Figure 5:**
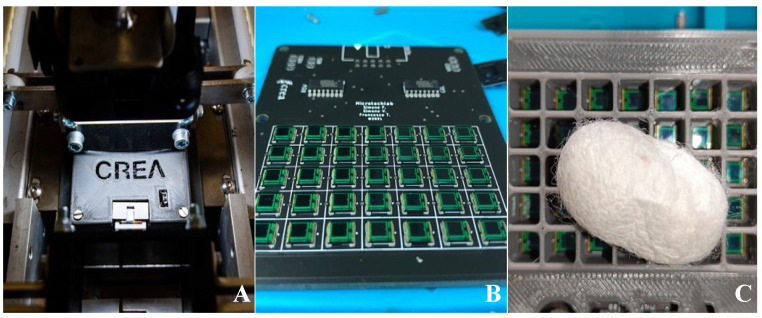
Light sensor in its case mounted on the prototype (**A**), assembled PCB of the light sensor (**B**) and positioning example of the cocoon over the matrix of light sensors (**C**).

This custom-made sensor is designed to read the light that is projected from a small LED panel placed above the cocoons. The sensor’s matrix external box was made with a 3D printer, as was the box for the Arduino and power management logic board, and was designed to fit in the specific mounting space; moreover, single sensors were isolated among themselves by a 3D-printed grille placed on the matrix itself. This design was adopted to prevent light leaks between sensors, because this behavior could cause false readings (Figure 5). The concept for developing this innovative sensor was based on two patents [22,23].

In detail, the sensor was based on the TEMD5510FX01 photodiode arranged matrix of 7 × 5 units, covering about 1749 mm^2^ of surface. The Arduino Portenta H7 was then able to read the voltage given by every photodiode using a single channel of the ADC working at 10-bit resolution (1024 ADC step values). This setup, which involved only one ADC, was designed to save time during the acquisition process and was implemented by using two fast 74HC4051D multiplexers made by Nexperia driven by four control busses each.

Every one of the 35 sensor readings was then performed by selecting first the row and then the column of the desired “pixel” using the control busses and eventually reading the Arduino Portenta’s ADC to store the value afterwards. Every pixel’s value was then saved in a vector inside Arduino’s flash memory and later wrote on the designated ModBus registers located inside the main PC.

The 35 signals obtained by the photodiode matrix were aligned, standardized through the Z-score and processed with a logistic regression algorithm for the 366 cocoons. The sample dataset was divided into two subsets: the training (80%) and test (20%) sets. The percentage of correct classification was extracted, and the results validated through cross validation averaging over 100 iterations.

### 2.6. Integration Model on Main PC

The software operated in two modes. The first was the acquisition of images from the camera and the matrix photodiode sensor to sample the cocoons for the construction of the model. The images from the three GigE Vision Cameras were stored on a disk, a folder for each camera, in the bmp format. The information from the photodiode sensor was stored in CSV format, in its folder. Each file was stored reporting the acquisition timestamp, the name of the camera and a progressive number that identified the acquisition, necessary to trace a cocoon in the sequence: shape and size, top outside stained, lower bottom outside stained and dead cocoons.

The second operating mode was the selection of the cocoons, with the use of the models described in points 2.2, 2.3 and 2.4, which were integrated into the software, exporting a library, one for each model, using the Matlab compiler. The image from the camera applied the relative algorithm of the model, returning if the cocoon was fit or not, for its related class. This procedure was applied for all the three cameras, except for the photodiode sensor, whose model was applied internally to the software without the use of external libraries, implementing a method that calculates the formula with the relative weights generated by the model.

## 3. Results

The prototype showed different performances according to the various selection parameters considered. At the beginning of the testing phase, due to the lightness of the cocoons and to avoid the excessive movement of the cocoons in their slot, which might, in turn, cause some alterations in image acquisition, the production frequency of the system was only 30 cocoons per minute. Nevertheless, as the prototype has constantly been improved in terms of hardware and software, at the moment the optimizations and tests carried out allowed the system to reach a frequency up to 80 cocoons per minute.

**Figure 6:**
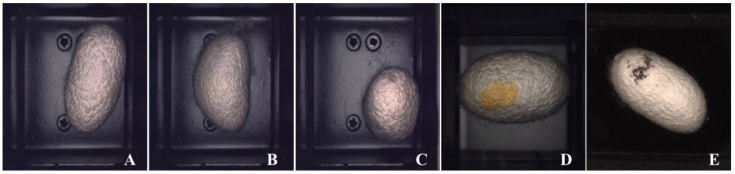
(**A**) Regular cocoon, (**B**) defective shape, (**C**) incorrect size due to positioning, (**D**) stained at the top (large orange spot), (**E**) stained at the bottom (black spot).

In cocoon size selection (oversize or undersize cocoons), the machinery reached optimum performances for oversize cocoons, classifying 87.8% of cocoons larger than 450 mm^2^ as oversized cocoons. However, the machine showed low performances (29.6% of correct classification) in selecting small-size cocoons (i.e., lower than 300 mm^2^), mainly due to the cocoons being vertically positioned inside their housing places (Figure 6). By eliminating the bad-positioned cocoons, the performance in detecting undersized cocoons increased to up to 90.0% correct classification. Results are in Table 2 according to the true positive percentage (defected cocoons marked as such) related to size selection.

**Figure 7:**
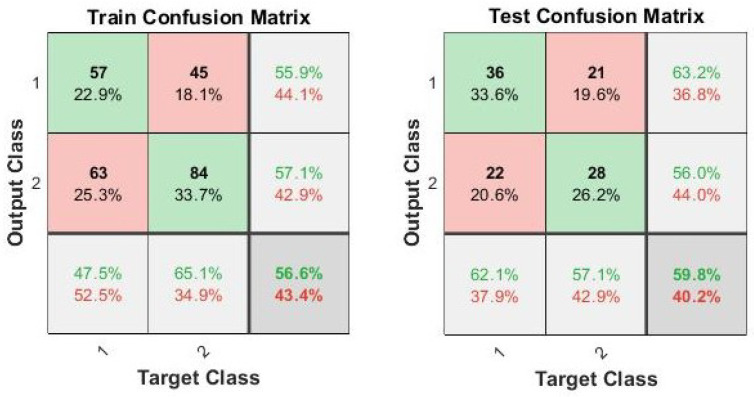
Confusion matrix of the cocoon-shape-selection procedure based on the logistic regression model as described in Section 2.3 (Class 1 stands for well-shaped cocoons; Class 2 stands for bad-shaped cocoons).

Well-shaped cocoons were selected through logistic regression applied on the Z-score of the EFA coefficients as input variables as described in Section 2.3. The correct classification ratio obtained for shape selection is 57% for the training set and 60% for the test set as shown in Figure 7. Cross validation with 100 iterations on the complete dataset was also performed with an average test accuracy (on the 100 iterations) of 54%.

The stained cocoon selection was done through the fixed-threshold processing of the top-view camera images, which showed a percentage of incorrect classification of 7.9% out of 852 unstained samples. This can be considered a good result if compared with the 14.6% error rate characterizing the manual procedure. The false-negative percentage (stained cocoons marked as unstained) was 22.9%. These failures in staining identification can be entirely attributed to the position of the spot on the side of the cocoon not directly caught by the camera due to its position. The fixed-threshold processing for the bottom camera showed a percentage of incorrect classification of 5.9% on 859 unstained cocoons and a false-negative percentage of 20.7%. This error was attributed to the position of the spot on the cocoon with respect to the camera even in this case. Results are summarized in Table 3 and Table 4.

**Figure 8:**
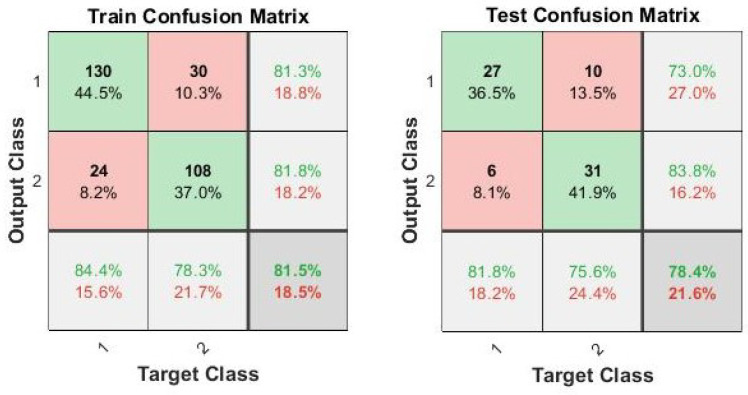
Confusion matrix of the dead-cocoon selection procedure based on the logistic regression model from the photodiode matrix (Class 1 stands for dead cocoons; Class 2 stands for alive cocoons).

The performances of the prototype in dead-cocoon selection based on the logistic regression model from the photodiode matrix are summarized in Figure 8. The correct classification ratio obtained for dead-cocoon selection is 81.5% on the training set and 78.4% on the test set. Cross validation with 100 steps was also performed with an average accuracy of 80.7% and 81.6% in training and testing, respectively.

In order to show the impact of each step on the overall process, the performances of the models used for the multi-step classification method are summarized in Table 5 in terms of overall accuracy and recall, defined respectively as:

$$A=\frac{Tp+Tn}{Tp+Tn+Fp+Fn};\;R=\frac{Tp}{Tp+Fn};$$

where *Tp* (*Tn*), and *Fp* (*Fn*) are the number of true-positive (negative) and false-positive (negative) cases. We consider positive cases undersized or oversized, not well-shaped, and stained and dead cocoons. The threshold values eventually used are also reported.

## 4. Discussion

Cocoon sorting is performed by farmers and perfected at the reeling plant, because of the importance of processing regular-size and perfectly undirtied cocoons in the reeling basins. This aspect is of basic relevance to obtain the 5A–6A (top level) [6]-quality silk required by the European silk textile industry. The prototype described in this paper was conceived by the authors to substitute for selection at the farmers’ site, which is very time-demanding and therefore critical in increasing production costs. The idea is that cocoon producers can transport their cocoons to the drying plant, where they can convey and desiccate their cocoons separately from other farmers (thanks to the traceability process established in the Serinnovation project) and according to the different quality classes (grades); the final aim is to calculate an adequate remuneration for each farmer, according to the accuracy of the work individually performed and, therefore, the quality of their eventual output [13], and also to transfer an already almost completely sorted product to the reeling plant to diminish selection work on cocoons to be reeled at the industrial place. The prototype was based on a patented idea [16].

The final output of the prototype should be compared to the manual output obtainable by subjective selection performed by the farmers. According to the sampling data of the sericultural laboratory of Padua, gained throughout the 5-year duration of the Serinnovation project, manual accuracy in cocoon sorting is rarely better than 80%, ranging from 75% for bad harvests and inexperienced farmers to 95% for good farmers and optimal harvesting seasons (authors’ unpublished data). In addition, the sorting efficiency of a trained operator in cocoon sorting is about 40–50 cocoons/minute (authors’ unpublished data). According to the real sorting efficiency of the prototype, the machine is a little bit slower than an experienced operator (30 vs. 45 cocoons/minute); however, the prototype has the chance of increasing its performance through a machine learning process, and the theorical speed is higher than the peak speed of the operator (80 vs. 50 cocoons per minute). Furthermore, it should be outlined that an operator is not capable of maintaining the full capacity and peak speed for many hours, and this is the reason why the farmers’ manual sorting process is imprecise. Therefore, both the number of resting pauses increases (therefore the average sorting speed decreases) or the efficiency in sorting diminishes considerably.

Therefore, the data obtained in Table 5 are quite encouraging by considering that the prototype’s performances can be increased through simple technical solutions.

These solutions are different considering the various quality parameters we took into account. In case of the errors in sorting cocoons according to their size, the failure of the system is almost always related to undersized cocoons when their largest diameter is not aligned to the longest axes of the cradles; this happens when they fall into the cradle in a vertical position rather than lying down. The possible solution may be to rely on two passages or to improve the cradle mouth so that cocoons are obliged to be better oriented in the same cradle. However, at the moment, 90% success for this parameter can be considered quite a good achievement. The prototype showed quite a poor performance in shape classification. This section of the machine is more difficult to set, as cocoons are not perfectly the same in their shape but are more or less rounded at tips and more or less constricted in the middle; in addition, their surface’s evenness is also dependent on many environmental factors and is rarely completely homogenous.

Moreover, the difficulty of sourcing bad-shaped cocoons resulted in a great sample imbalance with dramatic drawbacks on the model accuracy. The latter point can be overcome by collecting more samples and re-training the model. Luckily enough, only a very irregular cocoon shape affects the reeling process. Nevertheless, even in this case, the success ratio of cocoon sorting can be improved by improving lighting and making the background color more uniform (for example, by using a contrasting color). Concerning stained cocoons, the error ratio is quite low and acceptable, although this criticality can be almost eliminated, especially for the bottom camera, by changing the illumination conditions. The live vs. dead pupae selection in the cocoons is the most critical point, as supposed, for which an innovative and unique sensor was specifically designed. Further improvements can be obtained by reducing the light leakage by drastically reducing the height of the grid supporting cocoons and intensifying the illumination; in addition, to increase the sensitivity of the sensor, a higher-resolution ADC can be adopted. Unfortunately, cocoons with dead pupae in the reeling basin greatly affect the general reelability performances and the quality of the overall silk filament by impacting water cleanliness and therefore the other non-defective cocoons reeled together with the defective ones. This point appears, therefore, to be the most prominent issue on which to focus the next research efforts.

The concept on which this prototype is based is to combine cameras and classic approaches with custom sensors specifically realized from patents [22,23].

Generally speaking, this first prototype permitted the study of the patented idea in more detail and to individuate practical aspects on which further research is advisable.

## 5. Conclusions

Silk textile processing represents an agro-based industry engaged in manufacturing primary (cocoons) and secondary (silk skeins) products of agriculture. Like other industrial chains, it needs the standardization of the raw material to obtain high-quality goods for consumers. As cocoons are obtained through a biological activity from living organisms, they cannot be perfectly homogenous, and farmers should intervene in their selection through a high-labor and cost-demanding manual method. The mechanization of this operation can lower the production costs consistently and contribute to increasing the quality of the obtained silk. Therefore, the prototype described in this paper might be enlisted among the current efforts in reintroducing sericultural activity in Europe through regional and European projects based on the transfer of research innovations into practice and aimed at increasing the added value of the fiber intended for use in the textile luxury market or new smart applications. This prototype is a good workbench for future applications developed with new sensors or new AI algorithms, and it offers a chance to consistently improve the actual techniques used in cocoon sorting. Moreover, From the performance perspective, is possible to improve the accuracy of the machine, increasing the length of the belt, thus adding more control stations for cocoons processing. The speed of the selection process can be increased by adding one or more machines in parallel, all synced with each other, leading to a higher cocoons-per-minute rate.

## Figures and Tables

**Table 1:** HSB threshold values for top- and bottom-camera-position images.

Cameras	H	S	B
**Camera 1 Top**	0–80	60–255	0–255
**Camera 2 Bottom**	0–255	220–255	0–255

**Table 2:** The true positive percentage (defective cocoons marked as such).

Parameters	True Positive Percentage (%)
**Oversized cocoons (larger than 450 mm^2^)**	87.8
**Undersized cocoons (smaller than 300 mm^2^)**	29.6
**Undersized cocoons with no vertically positioned ones**	90.0

**Table 3:** Confusion matrix for the top-view camera.

		Target Class	
		Unstained	Stained
**Output class**	**Unstained**	785	85
	**Stained**	67	287

**Table 4:** Confusion matrix for the bottom-view camera.

		Target Class	
		Unstained	Stained
**Output class**	**Unstained**	808	77
	**Stained**	51	294

**Table 5:** Threshold values eventually used for discrimination, overall accuracy, and recall (see eq.) of the cocoon sorting prototype for the different parameters considered in this study.

Parameters	Threshold	Recall	Overall Accuracy (%)
**Size**	81,900 px^2^ < Size < 124,500 px^2^	0.57	90.0
**Shape training set**	-	0.65	56.6
**Shape test set**	-	0.57	59.8
**Stained top**	144 px^2^	0.77	87.6
**Stained bottom**	144 px^2^	0.79	89.6
**Alive–dead training set**	-	0.84	81.5
**Alive–dead test set**	-	0.82	78.4
